# Nitidine chloride inhibits hepatic cancer growth via modulation of multiple signaling pathways

**DOI:** 10.1186/1471-2407-14-729

**Published:** 2014-09-30

**Authors:** Jiumao Lin, Aling Shen, Hongwei Chen, Jun Liao, Teng Xu, Liya Liu, Jing Lin, Jun Peng

**Affiliations:** Academy of Integrative Medicine Biomedical Research Center, Fujian University of Traditional Chinese Medicine, Fuzhou, Fujian 350122 China; Fujian Key Laboratory of Integrative Medicine on Geriatrics, Fujian University of Traditional Chinese Medicine, Fuzhou, Fujian 350122 China; Department of Acupuncture and Moxa and Tuina, Fujian University of Traditional Chinese Medicine, Fuzhou, Fujian 350122 China

**Keywords:** Nitidine chloride, Hepatic cancer, HepG2 cells, Signaling transduction pathways, Cellular proliferation and apoptosis, Tumor angiogenesis

## Abstract

**Background:**

The development of hepatic cancer is tightly regulated by multiple intracellular signaling pathways. Therefore, most currently-used anti-tumor agents, which typically target single intracellular pathway, might not always be therapeutically effective. Additionally, long-term use of these agents probably generates drug resistance and unacceptable adverse effects. These problems increase the necessity for the development of new chemotherapeutic approaches. Nitidine chloride (NC), a natural benzophenanthridine alkaloid, has been shown to inhibit cancer growth via induction of cell apoptosis and suppression of cancer angiogenesis. But the precise mechanisms of its tumorcidal activity are not well understood.

**Methods:**

To further elucidate the precise mechanisms of its anti-tumor activity, using a hepatic cancer mouse xenograft model, the human hepatic cancer cell lines (HepG2, HCCLM3, Huh7), and umbilical vein endothelial cells (HUVEC), here we evaluate the effect of NC on tumor growth *in vivo* and *in vitro* and investigated the underlying molecular mechanisms.

**Results:**

We found that NC treatment resulted in significant decrease in tumor volume and tumor weight respectively, but didn’t affect body weight changes. Additionally, NC treatment dose- and time-dependently reduced the cell viability of all three hepatic cell lines. Moreover, NC suppressed the activation of STAT3, ERK and SHH pathways; and altered the expression of critical target genes including Bcl-2, Bax, Cyclin D1, CDK4, VEGF-A and VEGFR2. These molecular effects resulted in the promotion of apoptosis, inhibition of cell proliferation and tumor angiogenesis.

**Conclusions:**

Our findings suggest that NC possesses a broad range of anti-cancer activities due to its ability to affect multiple intracellular targets, suggesting that NC could be a novel multi-potent therapeutic agent for the treatment of hepatic cancer and other cancers.

## Background

Primary hepatic cancer or liver cancer is the sixth most commom cancer globally and the second leading cause of cancer-related death [1–5]. The most frequent hepatic cancer is hepatocellular carcinoma (HCC), accounting for approximately 75% of all primary liver cancers. Another type of liver cancer is hepatoblastoma (HBL), which is specifically formed by immature liver cells and primarily develops in children. To date, chemotherapy remains one of the major non-surgical therapeutic approaches for patients with advanced hepatic cancer [6]. However, due to drug resistance, systemic chemotherapy produces a disappointing low response rate, ranging between 10%-15% [7]. Moreover, many currently used anti-cancer agents have potent cytotoxic effects in normal cells [8]. These problems limit the effectiveness of current HCC chemotherapy, thus increasing the necessity for the development of new chemotherapeutic approaches.

The mechanisms underlying pathogenesis and development of HCC are complex and heterogeneous, involving multiple cellular signaling pathways including signal transducer and activator of transcription 3 (STAT3), Sonic Hedgehog (SHH) and extracellular regulated protein kinases (ERK). STAT3 plays an essential role in cell survival, proliferation and angiogenesis [9]. After activation via phosphorylation, STAT3 proteins in the cytoplasm dimerize and translocate to the nucleus where they regulate the expression of critical genes involved in cancer progression. Constitutive activation of STAT3 is strongly associated with cancer development and commonly suggests a poor prognosis [10, 11]. Aberrant activation of SHH is highly correlated with various human cancers [12–14]. SHH signaling activation is initiated at the cell surface by binding of SHH ligand to the transmembrane receptor Patched (Ptc), resulting in the release of Ptc-mediated suppression of Smoothened (Smo). Smo subsequently activates the Gli family of transcription factors that regulate the expression of various HH target genes [15–17]. Extracellularsignal regulated kinase (ERK) signaling is one of the major cell-survival and proliferation pathways. As a major subfamily member of Mitogen-activated protein kinases (MAPKs), activation of ERK is regulated by a central three-tiered kinase core consisting of MAPK kinase kinase (e.g., Raf), MAPK kinase (e.g., MEK), and MAPK (e.g., ERK); wherein Raf phosphorylates MEK which in turn phosphorylates and activates ERK [18]. By altering the levels and activities of transcription factors, activation of ERK pathway regulates the expression of various genes mediating cell apoptosis, proliferation and angiogenesis [19, 20]. These molecular pathways described above modulate the expression of key genes involved in the regulation of cell proliferation, apoptosis, and angiogenesis and are participants in the processes of induction, progression, and metastasis of hepatic cancer. Thus, each serves as a potential target for novel chemotherapeutics.

Natural products have received recent interest in discovery of novel anti-cancer therapeutic agents as they have relatively few side effects and have long been used as alternative remedies for a variety of diseases including cancer [21, 22]. Therefore, identifying naturally occurring agents is a promising approach for anticancer treatment. Nitidine chloride, a natural benzophenanthridine alkaloid, is a major active compound present in a well-known traditional Chinese medicinal herb *Zanthoxylum nitidum (Roxb)* DC. Previous studies found that NC has antifungal, anti-inflammatory and analgesic activities [23, 24]. Recently it has been shown that NC inhibits the growth of many human cancer cells via induction of cell apoptosis [25]. Moreover, Chen et al. reported that NC can suppress gastric cancer angiogenesis by inhibition of STAT3 pathway [26], and we previously reported that the NC is able to inhibit hepoatocellular carcinoma growth via modulation of JAK1/STAT3 pathway [27]. In order to further elucidate the mechanism of tumorcidal activity of NC, in the present study we evaluated its effect on hepatic cancer growth *in vivo* and *in vitro*, and investigated the underlying molecular mechanisms.

## Methods

### Materials and reagents

Nitidine Chloride (NC, purity >98%) was provided from Institute of Sichuan Xianxin Biochemical Technology (Sichuan, China). Matrigel was provided by Becton Dickinson (San Jose, CA, USA). Roswell Park Memorial Institute Medium 1640 (RPMI 1640), Dulbecco’s modified Eagle’s medium (DMEM), fetal bovine serum (FBS), penicillin-streptomycin, trypsin-EDTA, 5,5’,6,6’-tetrachloro-1,1’,3,3’-tetraethyl-benzimidazol-carbocyanine iodide (JC-1), were purchased from Invitrogen (Grand Island, NY, USA). The *In Vitro* Angiogenesis Assay Kit was purchased from Millipore (Billerica, MA, USA). A fluorescein isothiocyanate (FITC)-conjugated annexin V apoptosis detection kit was provided by Becton Dickinson (San Jose, CA, USA). TUNEL assay kit (TumorTACS *in situ*) was purchased from R&D Systems (Minneapolis, MN, USA). All antibodies were purchased from Cell Signaling Technology (Beverly, MA, USA). BCA Protein Assay Kit was purchased from Tiangen Biotech Co., Ltd. (Beijing, China). Cignal STAT3 Reporter (luc) Kit was obtained from SABiosciences, QIAGEN company (Hilden, Germany). All other chemicals, unless otherwise stated, were obtained from Sigma-Aldrich (St. Louis, MO, USA).

### Cell culture

Human hepatic cancer cell lines (HepG2, HCCLM3 and Huh7) and human umbilical vein endothelial cells (HUVECs) were purchased from Xiangya Cell Center (Hunan, China). HepG2 cells and HUVECs were grown in DMEM and RPMI 1640, respectively. Both DMEM and RPMI 1640 were supplemented with 10% (v/v) FBS, 100 units/ml penicillin, and 100 μg/ml streptomycin.

### Animals

Male BALB/C athymic nude mice (with an initial body weight of 20–22 g) were obtained from Shanghai SLAC Laboratory Animal Co., Ltd. (Shanghai, China) and housed under pathogen-free conditions with controlled temperature (22°C), humidity, and a 12 hour light/dark cycle. Food and water were given *ad libitum* throughout the experiment. All animal treatments were performed strictly in accordance with international ethical guidelines and the National Institutes of Health Guide concerning the Care and Use of Laboratory Animals. The experiments were approved by the Institutional Animal Care and Use Committee of Fujian University of Traditional Chinese Medicine.

### In vivo nude mice xenograft study

Hepatic cancer xenograft mice were produced with HepG2 cells. The cells were grown in culture and then detached by trypsinization, washed, and resuspended in serum-free DMEM. Resuspended cells (5 × 10^6^) mixed with Matrigel (1:1) were subcutaneously injected into the right flank of mice to initiate tumor growth. At 5 days following xenograft implantation (tumor size approximately 3 mm in diameter), mice were randomized into two groups (*n* = 10) and treated with 4.5 mg/kg of NC (dissolved in saline) or saline daily by intraperitoneal injection, 6 days a week for 18 days. Body weight and tumor size were measured. Tumor size was determined by measuring the major (L) and minor (W) diameter with a caliper. The tumor volume was calculated according to the following formula: tumor volume = π/6 × L × W^2^. At the end of the experiment, the animals were anaesthetized and tumors were excised and weighed.

### Cell viability evaluation by MTT assay

NC was dissolved in DMSO and diluted to working concentrations with culture medium. The final concentration of DMSO in the medium for all cell-based experiments was 0.1%. Cells (HepG2, HCCLM3, Huh7 cells or HUVECs) were seeded into 96-well plates at a density of 1.0 × 10^4^ cells/well in 0.1 ml medium. 24 h later, cells were treated with various concentrations of NC for different time periods. After NC treatment, 10 μl MTT (5 mg/ml in phosphate buffered saline (PBS)) were added to each well, and the samples were incubated for an additional 4 h at 37°C. The purple-blue MTT formazan precipitate was dissolved in 100 μl DMSO. Absorbance was measured at 570 nm using an ELISA reader (BioTek, Model EXL800, USA).

### Colony formation assay

HepG2 cells from different treated groups were seeded in 6-well plates with a density of 200 cells per well for 7 days. The medium was discarded and each well was washed twice with PBS carefully. The colonies were fixed in methanol for 20 min and then stained with Giemsa staining solution. The number of colonies with ≥50 cells was counted and colony forming efficiency was calculated (Percentage of colonies = Number of colonies formed/Number of cells inoculated × 100%).

### Cell cycle analysis

Cell cycle analysis was carried out by flow cytometry using FACS analysis with propidium iodide (PI) staining. HepG2 cells were treated with various concentrations of NC for 24 h, harvested, adjusted to a concentration of 1 × 10^6^ cells/ml, and fixed in 70% ethanol at 4°C overnight. The fixed cells were washed twice with cold PBS, and incubated for 30 min with RNase (8 μg/ml) and PI (10 μg/ml). The fluorescent signal was detected through the FL2 channel and the proportion of DNA in different phases was analyzed using ModfitLT Version 3.0 (Verity Software House, Topsham).

### Apoptosis detection in HepG2 cells by flow cytometry analysis with annexin V/PI staining

After incubation with various concentrations of NC, apoptosis of HepG2 cells were determined by flow cytometry using a fluorescence-activated cell sorting (FACS) caliber (Becton Dickinson, CA, USA) and Annexin V-fluorescein isothiocyanate (FITC)/propidium iodide (PI) kit (Becton Dickinson). Staining was performed according to the manufacturer’s instructions. The percentage of cells in early apoptosis was calculated by Annexin V-positivity and PI-negativity, and the percentage of cells in late apoptosis was calculated by Annexin V-positivity and PI-positivity.

### Measurement of mitochonrial membrane potential (Δψm) by flow cytometry analysis with JC-1 staining

JC-1 is a cationic dye that exhibits potential mitochondria-dependent accumulation, indicated by a fluorescence emission shift from green to red, which thus can be used as an indicator of mitochondrial potential. In this experiment, 1×10^6^ treated HepG2 cells were resuspended after trypsinization in 1 ml of medium and incubated with 10 μg/ml of JC-1 (Invitrogen) at 37°C, 5% CO_2_, for 30 min. Both red and green fluorescence emissions were analyzed by flow cytometry after JC-1 staining.

### Apoptosis detection in hepatic tumor tissues by TUNEL staining

Six tumors were randomly selected from NC-treatment or control groups. Tumor tissues were fixed in 10% formaldehyde for 12 h, paraffin-embedded and then sectioned into 4-μm-thick slides. Samples were analyzed by TUNEL staining using TumorTACS *in situ* kit (R&D Systems). Apoptotic cells were counted as DAB-positive cells (brown stained) at five arbitrarily selected microscopic fields at a magnification of 400×. TUNEL-positive cells were counted as a percentage of the total cells.

### Immunohistochemistical analysis of hepatic tumor tissues

Six tumors were randomly selected from NC-treatment or control groups. Tumor tissues were fixed in 10% formaldehyde for 12 h, paraffin-embedded, sectioned, and placed on slides. The slides were subjected to antigen retrieval and endogenous peroxidase activity was quenched with hydrogen peroxide. Non-specific binding was blocked with normal serum in PBS (0.1% Tween 20). Rabbit polyclonal antibodies against Ki-67, CD31, Shh and Gli-1 (all in 1:200 dilution, Santa Cruz Biotechnology) were used to detect the relevant proteins. The binding of the primary antibody was demonstrated with a biotinylated secondary antibody, horseradish peroxidase (HRP)-conjugated streptavidin (Dako), and diamino-benzidine (DAB) as the chromogen. The tissues were counterstained with diluted Harris hematoxylin. After staining, five high-power fields (at magnification of 400×) were randomly selected in each slide. The proportion of positive cells in each field was determined using the true color multi-functional cell image analysis management system (Image-Pro Plus, Media Cybernetics, USA). To control for nonspecific staining, PBS was used to replace the primary antibody as a negative control.

### Tube formation assay of HUVECs

HUVEC tube formation was examined using the ECMatrix assay kit (Millipore) following the manufacturer’s instructions. Briefly, confluent HUVECs were harvested and diluted (1 × 10^4^ cells) in 50 μl of medium containing various concentrations of NC. The harvested cells were seeded with ECMatrix gel (1:1 v/v) into 96-well plates and incubated for 9 h at 37°C. The cells were photographed using phase-contrast inverted microscopy at a magnification of 100 ×.

### RT-PCR analysis

Total RNA was isolated from tumor tissues (three tumors were randomly selected from NC-treatment or control groups) or HepG2 cells and HUVECs with TriZol Reagent (Invitrogen). Oligo (dT)-primed RNA (1 μg) was reverse-transcribed with SuperScript II reverse transcriptase (Promega) according to the manufacturer’s instructions. The obtained cDNA was used to determine the mRNA amount of Cyclin D1, CDK4, Bcl-2, Bax, SHH, Gli-1, VEGF and VEGFR2 by PCR with Taq DNA polymerase (Fermentas). GAPDH was used as an internal control. Samples were analyzed by gel electrophoresis (1.5% agarose). The DNA bands were examined using a Gel Documentation System (BioRad, Model Gel Doc XR+, USA).

### Western blotting analysis

Three tumors were randomly selected from NC-treatment or control groups. Tumor tissues were homogenized in nondenaturing lysis buffer and centrifuged at 14,000 × g for 15 min. Protein concentrations of the clarified supernatants were determined by BCA protein assay. HepG2 cells or HUVECs (2.5 × 10^5^) in 5 ml medium were seeded into 25 cm^2^ flasks and treated with the indicated concentrations of NC for 24 h. Treated cells were lysed in mammalian cell lysis buffer (M-PER, Thermo Scientific, Rockford, IL, USA) containing protease (EMD Biosciences) and phosphatase inhibitor (Sigma-Aldrich) cocktails, and centrifuged at 14,000 × g for 15 min. Protein concentrations in cell lysate supernatants were determined by BCA protein assay. Equal amounts of protein from each tumor or cell lysate were resolved on 12% Tris-glycine gels and transferred onto PVDF membranes. The membranes were blocked for 2 h with 5% nonfat dry milk and incubated with the desired primary antibody directed against STAT3, pSTAT3, ERK, pERK, Cyclin D1, CDK4, Bcl-2, Bax, VEGF, VEGFR2 or β-actin (all in 1:1000 dilutions) overnight at 4°C. Appropriate HRP-conjugated secondary antibodies with chemiluminescence detection were used to image the antibody-detected proteins.

### Luciferase gene reporter assay

HepG cells were seeded into 96-well plates at a density of 1 × 10^4^ cells/well in 0.1 ml complete DMEM until about 50% confluency and then continuously cultured in FBS- and antibiotics-free medium overnight. Cells were transfected with a mixture of inducible STAT3-responsive firefly luciferase construct and constitutively expressing Renilla luciferase construct using Lipofectamine™ LTX with PLUS™ Reagent. 6 h after transfection the medium was changed back into DMEM complete with FBS, penicillin and streptomycin. After 24 hours of transfection, cells were treated with various contractions of NC for 1 h followed by IL-6 for another 24 h. Cell extracts were prepared and analyzed using Promega Dural Luciferase Reporter Assay System according to the manufacturer’s instruction. The measured firefly luciferase activity was normalized to the activity of Renilla luciferase in the same well.

### Statistical analysis

Data were presented as mean ± SD for the indicated number of independently performed experiments. Statistical analysis was carried out with Student’s *t*-test and ANOVA. Differences with *P* < 0.05 were considered to be statistically significant.

## Results and discussion

### NC inhibits hepatic cancer growth *in vitro*and *in vivo*

**Figure 1:**
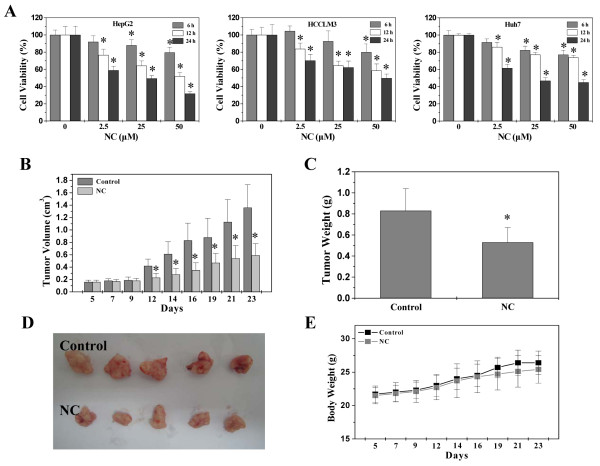
**Effect of NC on hepatic cancer growth** ***in vitro*** **and** ***in vivo*** **. (A)** Viability of HepG2, HCCLM3 or Huh7 cells was determined by the MTT assay after cells were treated with the different concentrations of NC for the indicated time periods. *P < 0.05, versus control cells. **(B-E)** In vivo study. After tumor development, mice were randomized into two groups (*n* = 10) and treated with 4.5 mg/kg of NC or saline daily by intraperitoneal injection, 6 days a week for 18 days. Tumor volume **(B)**, tumor weight **(C)** and body weight **(E)** were measured. **(D)** Representative images for tumors. Data shown are averages with S.D. (error bars) from 10 individual mouse in each group. *P < 0.01, versus controls.

We evaluated the *in vitro* anti-cancer effect of NC by examining the viability of three human hepatic cell lines (HepG2, HCCLM3 and Huh7) using MTT assay. As shown in Figure 1A, treatment with NC dose- and time-dependently reduced the viability of all three hepatic cell lines (*P* < 0.05). The *in vivo* anti-tumor activity of NC was evaluated by comparing the tumor weight and volume in treated and control HepG2 xenograft mice, while its adverse effects were determined by measuring changes in body weight. As shown in Figure 1B-D, NC treatment resulted in 56.62% and 36.14% decrease in tumor volume and tumor weight respectively, as compared to control (*P* < 0.01). However, administration of NC had no effect on body weight changes during the course of the study (Figure 1E). Taken together, these data suggest that NC is effective in suppressing liver tumor growth both *in vivo* and *in vitro*, without apparent signs of toxicity.

### NC inhibits hepatic cancer cell proliferation via G1/S cell cycle arrest

**Figure 2:**
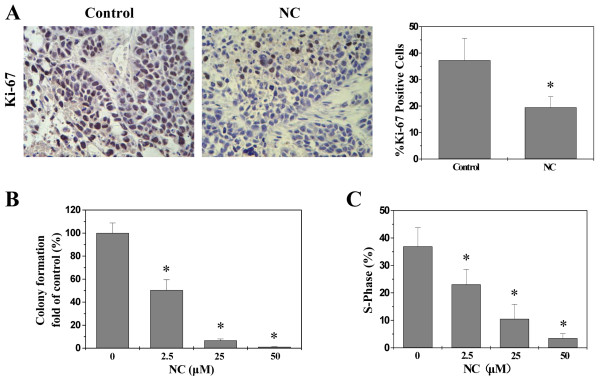
**Effect of NC on cell proliferation in hepatic cancer xenograft mice and HepG2 cells. (A)** Ki-67 assay in tumor tissues (400 ×). Data shown are averages with S.D. (error bars) from 6 individual mouse in each group. *P < 0.01, versus controls. **(B)** HepG2 cell colony formation assay. The data were normalized to the viability or survival of control cells (100%, treated with 0.1% DMSO vehicle). **(C)** HepG2 cell cycle was analyzed by FACS and the proportion of cells in S-phase was calculated. Data from panels **B**-**D** are averages with S.D. (error bars) from at least three independent experiments. *P < 0.01, versus control cells.

Cancer cells are characterized by an uncontrolled increase in cell proliferation; we therefore determined the proliferative activity of NC. Cell proliferation in tumor tissues was determined via immunohistochemical staining (IHC) for Ki-67, a proliferation marker that is specifically expressed in proliferating cell nuclei. As shown in Figure 2A, the percentage of Ki-67 positive cells in tumor tissues from control and NC-treated xenograft mice was 37.3 ± 8.3% and 19.5 ± 4.0%, respectively (*P* < 0.01). The *in vitro* proliferation of HepG2 cells was determined by colony formation assay. As shown in Figure 2B, treatment with 2.5, 25 and 50 μM of NC for 24 h reduced the cell survival rate by 50%, 93% and 99% compared to untreated control cells (*P* < 0.01). Thus, NC can inhibit hepatic cancer cell proliferation both *in vivo* and *in vitro*.

**Figure 3:**
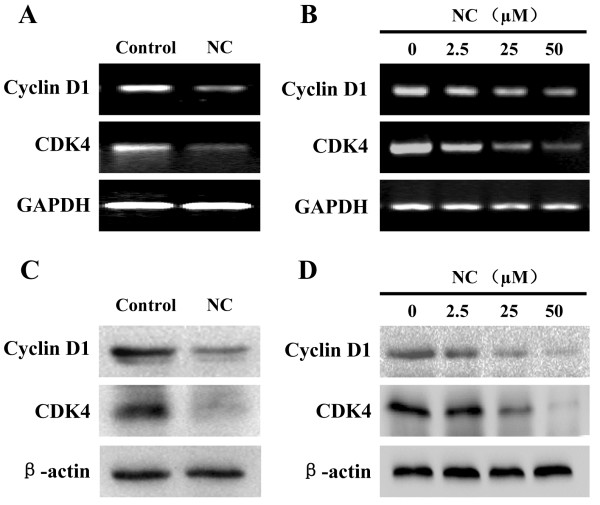
**Effect of NC on the expression of Cyclin D1 and CDK4 in hepatic cancer xenograft mice and HepG2 cells.** The mRNA levels of Cyclin D1 and CDK4 in tumor tissues **(A)** or HepG2 cells **(B)** were determined by RT-PCR. The protein expression levels of Cyclin D1 and CDK4 in tumor tissues **(C)** or HepG2 cells **(D)** were determined by Western blotting. GAPDH and β-actin were used as the internal controls for the RT-PCR or Western blotting, respectively. Images are representatives of 3 individual mouse in each group or of three independent cell-based experiments.

Eukaryotic cell proliferation is primarily regulated by cell cycle. G1/S transition is one of the major cell cycle checkpoints [28], which is responsible for initiation and completion of DNA replication. G1/S progression is strongly regulated by Cyclin D1 that exerts its function via forming an active complex with its CDK major catalytic partners (CDK4/6) [29]. An unchecked or hyperactivated Cyclin D1/CDK4 complex often leads to uncontrolled cell division and malignancy [30–32]. The effect of NC on the G1 to S progression in HepG2 cells was investigated via PI staining followed by FACS analysis. We found that the percentage of HepG2 cells in S-phase following NC treatment was decreased in a dose-dependent manner (Figure 2C, *P* < 0.01). In addition, data from RT-PCR and Western Blot analysis showed that NC treatment significantly reduced the mRNA and protein levels of pro-proliferative Cyclin D1 and CKD4 both in hepatic tumor tissues and HepG2 cells (Figure 3). These data together suggest that NC inhibits hepatic cancer cell proliferation through blockade of G1-S progression and the modulation of the expression of cell cycle-regulatory genes.

### NC induces hepatic cancer cell apoptosis via activation of the mitochondrion-dependent pathway

**Figure 4:**
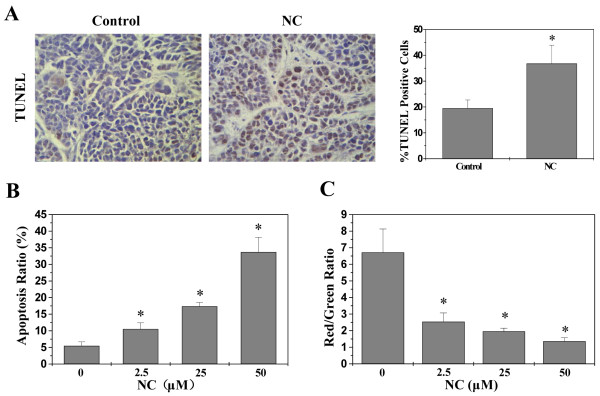
**Effect of NC on cell apoptosis in hepatic cancer xenograft mice and HepG2 cells. (A)** TUNEL assay in tumor tissues (400 ×). Data shown are averages with S.D. (error bars) from 6 individual mouse in each group. *P < 0.01, versus controls. **(B)** Apoptosis of HepG2 cells were quantification of FACS analysis. **(C)** Quantitative analysis of the JC-1 red/green fluorescent intensity ratio. The data from C and E shown are averages with S.D. (error bars) from three independent experiments. *P < 0.05, versus controls.

Apoptosis is crucial for animal development and tissue homeostasis. Disturbed regulation of this vital process represents a major causative factor in tumorigenesis. Promoting cell apoptosis therefore has been a major focus in the development of anti-cancer therapies. In the present study we evaluated apoptosis in tumors via TUNEL assay; and we found that the percentage of TUNEL-positive cells in tumors from NC-treated mice or controls was 36.8 ± 7.1% or 19.5 ± 3.2%, respectively (Figure 4A, *P* < 0.01), suggesting that NC promotes cell apoptosis in tumor tissues. The apoptosis of HepG2 cells was determined via Annexin-V/PI staining followed by FACS analysis. As shown in Figure 4B, the percent of cells undergoing either early or late apoptosis following treatment with 2.5, 25 or 50 μM of NC was 8.50%, 17.12% or 32.19%, whereas the apoptotic rate in untreated control cells was 5.48% (*P* < 0.05). Thus NC induces hepatic cancer cell apoptosis *in vitro* in a dose-dependent manner.

The mitochondrion-dependent pathway is the most common apoptotic pathway in vertebrate animal cells. Mitochondrial outer membrane permeabilization (MOMP) is a key commitment step in the induction of cellular apoptosis, since it is the point of convergence for a large variety of intracellular apoptotic signaling pathways leading to the release of many apoptogenic proteins from the mitochondrial intermembrane space. During the process of MOMP, the electrochemical gradient across the mitochondrial membrane collapses. Therefore, the loss of mitochondrial membrane potential is a hallmark for apoptosis. To investigate the mechanism of how NC induced HepG2 cell apoptosis, we used FACS analysis with JC-1 staining to examine the change of mitochondrial membrane potential in NC-treated HepG2 cells. The membrane-permeant JC-1 dye displays potential-dependent accumulation in mitochondria, indicated by a fluorescence emission shift from green (~525 nm) to red (~590 nm). Therefore, collapse of mitochondrial potentail during apoptosis is indicated by a decrease in the ratio of red/green fluorescence intensity. As shown in Figure 4C, after treatment with 0, 2.5, 25, 50 μM of NC the JC-1 red/green fluorescent ratio in HepG2 cells was 6.71 ± 1.52, 2.54 ± 0.53, 1.96 ± 0.19, 1.36 ± 0.22, respectively, suggesting that NC dose-dependently induces the loss of mitochondrial membrane potential in HepG2 cells.

**Figure 5:**
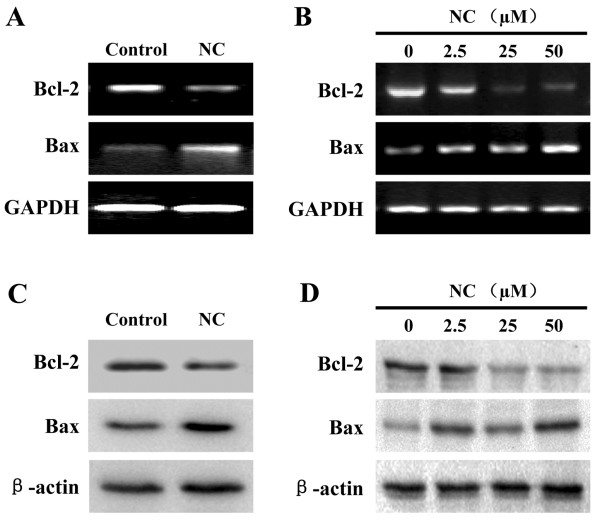
**Effect of NC on the expression of Bcl-2 and Bax in hepatic cancer xenograft mice and HepG2 cells.** The mRNA levels of Bcl-2 and Bax in tumor tissues **(A)** or HepG2 cells **(B)** were determined by RT-PCR. The protein expression levels of Bcl-2 and Bax in tumor tissues **(C)** or HepG2 cells **(D)** were determined by Western blotting. GAPDH and β-actin were used as the internal controls for the RT-PCR or Western blotting, respectively. Images are representatives of 3 individual mouse in each group or of three independent cell-based experiments.

Bcl-2 family proteins are key regulators of mitochondrion-mediated apoptosis, functioning as either suppressors such as Bcl-2, or promoters such as Bax. MOMP is thought to occur through the formation of pores in the mitochondria by pro-apoptotic Bax-like proteins, which can be inhibited by anti-apoptotic Bcl-2-like members. Therefore, the ratio of active anti- and pro-apoptotic Bcl-2 family members determines the fate of cells, and alteration of the ratio by aberrant expression of these proteins impairs the normal apoptotic program contributing to various apoptosis-related diseases including cancer [33]. Higher Bcl-2 to Bax ratios are commonly found in cancers, which not only confers a survival advantage to the cancer cells but also causes resistance to chemo- and radio-therapies. To further study the mechanism of NC’s pro-apoptotic activity, we performed RT-PCR and Western Blotting to respectively examine the mRNA and protein expression of Bcl-2 and Bax. As shown in Figure 5, NC treatment significantly reduced the mRNA and protein expression levels of anti-apoptotic Bcl-2 both in the tumor tissues and HepG2 cells, whereas those of pro-apoptotic Bax were significantly increased after NC treatment, suggesting that NC induces hepatic cancer cell apoptosis both *in vivo* and *in vitro* through an increase in the pro-apoptotic Bax/Bcl-2 ratio.

### NC inhibits hepatic tumor angiogenesis via suppressing the expression of VEGF-A and VEGFR2

**Figure 6:**
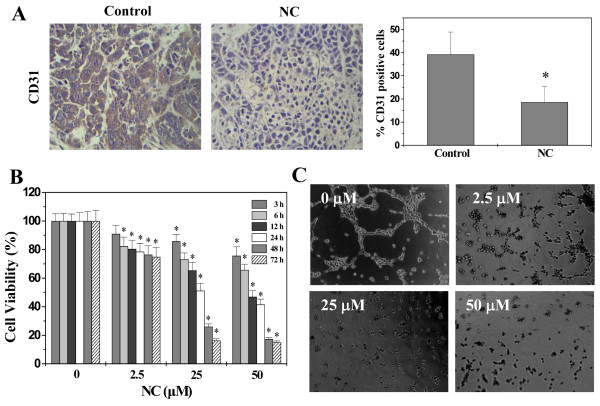
**Effect of NC on angiogenesis in hepatic cancer xenograft mice and HUVECs. (A)** CD31 assay in tumor tissues (400 ×). Data shown are averages with S.D. (error bars) from 6 individual mouse in each group. *P < 0.01, versus controls. **(B)** HUVEC’s viability was determined by the MTT assay after cells were treated with the various concentrations of NC for the indicated time periods. The data were normalized to the viability of control cells (100%, treated with 0.1% DMSO vehicle). Data are averages with S.D. (error bars) from at least three independent experiments. *P < 0.05, versus controls. **(C)** Tube formation assay in HUVEc (100 ×). The network-like structures were examined by phase-contrast microscopy. Images are representatives of three independent experiments.

Formation of new blood vessels via angiogenesis is critical for the development of solid tumors [34–37]. Initially, tumor cells obtain oxygen and nutrients from nearby blood vessels by simple passive diffusion. However, when tumor grows to certain size, oxygen delivery by diffusion is no longer sufficient, which causes tumor cells to induce the sprouting of new blood vessels from pre-existing vasculature, creating a blood supply system within solid tumor that is essential for continuous growth of tumor as well as providing an avenue for hematogenous metastasis [38, 39]. To determine the effect of NC on angiogenesis *in vivo*, we performed IHC for the expression of the endothelial cell–specific marker CD31 to examine intratumoral microvessel density (MVD). As shown in Figure 6A, the percentage of CD31-positive cells in NC-treated mice was significantly reduced (*P* < 0.01). The processes of angiogenesis include endothelial cell (EC) proliferation, migration, and alignment into tubular structures. To further evaluate the anti-angiogenic effect of NC we modeled each of these processes with HUVECs *in vitro*. As shown in Figure 6B, NC treatment dose- and time-dependently decreased the proliferation (viability) of HUVECs compared to untreated control cells (*P* < 0.05). Moreover, we examined NC’s effect on capillary tube formation of HUVECs using an extracellular matrix, in which cultured ECs rapidly align and form hollow tube-like structures. As shown in Figure 6C, untreated HUVECs formed elongated tube-like structures, whereas NC treatment resulted in a significant decrease in capillary tube formation. Collectively, these data suggest that inhibition of tumor angiogenesis by NC could have contributed to the inhibition of hepatic cancer growth.

**Figure 7:**
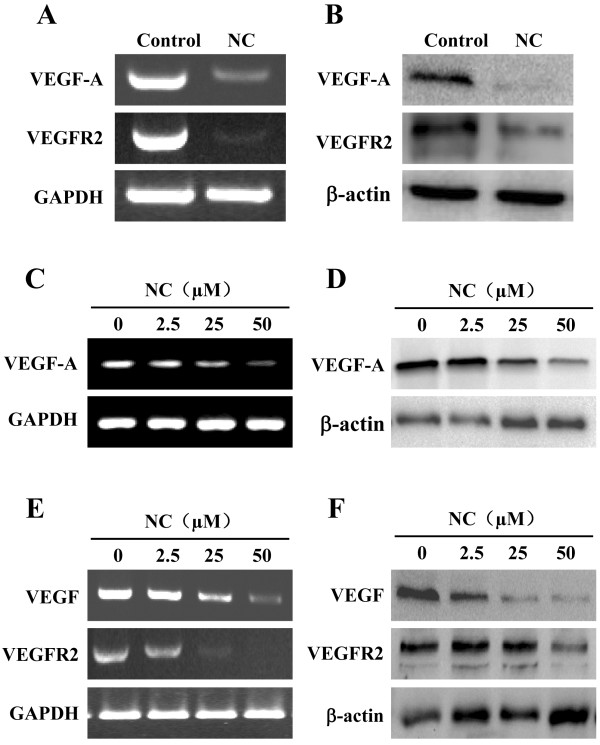
**Effect of NC on the expression of VEGF-A and VEGFR2.** The mRNA and protein expression levels of VEGF-A and VEGFR2 in tumor tissues **(A, B)** or in HepG2 cells **(C, D)** and HUVECs **(E, F)** were determined by RT-PCR and Western Blotting. GAPDH and β-actin were used as the internal controls for the RT-PCR or Western Blotting, respectively. Images are representatives of 3 individual mouse in each group or three independent cell-based experiments.

Induction of angiogenesis is mediated by a variety of molecules released by tumor cells [40]. Vascular endothelial growth factor A (VEGF-A) is considered one of the strongest angiogenic stimulators [41]. VEGF-A is highly expressed in a wide variety of human tumors, which has been associated with tumor progression, invasion and metastasis, and poorer survival and prognosis in patients [42, 43]. VEGF-A is secreted by tumor cells and endothelial cells and functions via paracrine and autocrine signaling pathways. When VEGF-A is secreted, it primarily binds to specific receptors located on vascular endothelial cells (EC) [44], which in turn triggers a tyrosine kinase signaling cascade that eventually induces angiogenesis [44, 45]. To explore the mechanism of NC’s anti-angiogenic activity, we determined its effect on the expression of VEGF-A and its specific receptor VEGFR2 both *in vivo* and *in vitro*. As shown in Figure 7A and B, NC treatment profoundly decreased mRNA and protein levels of VEGF-A and VEGFR2 in hepatic cancer xenograft tumor tissues. Similarly, NC dose-dependently reduced VEGF-A expression in HepG2 cells and HUVECs and also the expression of VEGFR-2 in HUVECs, at both transcriptional and translational levels (Figure 7C-F).

### NC suppresses STAT3, ERK and SHH pathways

**Figure 8:**
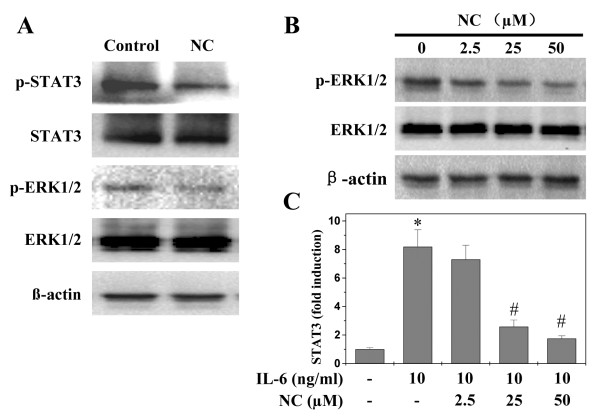
**Effect of NC on activation of STAT3 and ERK in hepatic cancer xenograft mice and HepG2 cells.** The level of STAT3 or ERK phosphorylation in tumor tissues **(A)** and ERK phosphorylation in HepG2 cells **(B)** was determined by Western blot. β-actin was used as the internal control. Data panels from **A**-**B** are representatives of 3 individual mouse in each group. **(C)** IL-6-mediated STAT3 transcriptional activity in HepG2 cells. The transcriptional activity of STAT3 was measured by DLR assay. Data are averages with S.D. (error bars) from at least three independent experiments. *P < 0.05, versus control cells; ^***#***^ *P* < 0.05, versus cells treated with IL-6 but without NC.

**Figure 9:**
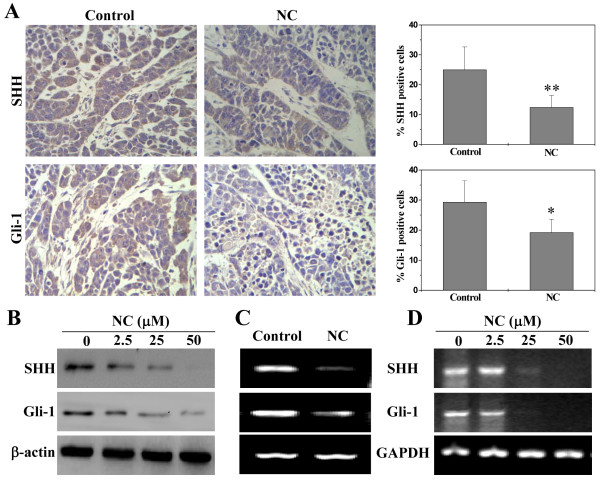
**Effect of NC on the activation of SHH pathway in hepatic cancer xenograft mice and HepG2 cells. (A)** Tumor tissues were processed for IHC for SHH and Gli-1. The photographs are representative images taken at a magnification of 400 ×. Quantification of IHC assay was represented as percentage of positively-stained cells. Data shown are averages with S.D. (error bars) from 6 individual mouse in each group. *P < 0.05; **P < 0.01, versus controls. **(B)** The protein expression levels of Shh and Gli-1 in HepG2 cells were determined by Western Blotting. β-actin was used as the internal control. The mRNA levels of SHH and Gli-1 in tumor tissues **(C)** and HepG2 cells **(D)** were determined by RT-PCR. GAPDH was used as the internal control. Images of Western Blotting and RT-PCR are representatives of 3 individual mouse in each group.

Cancer development is tightly regulated by multiple intracellular signaling pathways, including STAT3, ERK and SHH. Aberrant activation of these pathways alters the expression of various critical target genes mediating the processes of apoptosis, proliferation and angiogenesis. To further elucidate the mechanisms of anti-tumor activity of NC, we determined its effect on the activation of STAT3, ERK and SHH pathways. Activation of STAT3 and ERK is mediated by its phosphorylation, we therefore investigated STAT3 and ERK activation in HCC tumor tissues and/or HepG2 cells by Western Blot analysis using antibodies that recognize STAT3 or ERK phosphorylation. As shown in Figure 8A and B, NC treatment decreased the levels of phosphorylated STAT3 and ERK in tumors of xenograft mice and/or in HepG2 cells. The levels of non-phosphorylated STAT3 and ERK remained unchanged. To further explore the mechanisms whereby NC suppressed the activation of STAT3, we performed Dual Luciferase Reporter assay to examine STAT3 transcriptional activity in HepG2 cells. Results from Figure 8C showed that NC significantly and dose-dependently inhibited IL-6-stimulated increase of STAT3 transcriptional activity. We assessed the effect of NC on the expression of key mediators of SHH pathway in hepatic cancer xenograft tumors and HepG2 cells using IHC, Western Blot and RT-PCR analyses. As shown in Figure 9A and B, NC treatment significantly decreased the protein expression of SHH- or Gli-1-positive cells both in the hepatic cancer xenograft tumors and HepG2 cells. Data from RT-PCR showed that the pattern of mRNA expression was similar to their respective protein levels (Figure 9C and D). Taken together, these data suggest that NC significantly suppresses the activation of multiple hepatic cancer-related signaling pathways.

## Conclusions

In summary, here for the first time we demonstrate that Nitidine chloride possesses a broad range of anti-cancer activities due to its ability to affect multiple intracellular targets. Our findings suggest that NC could be a novel therapeutic agent for the treatment of hepatic cancer and other malignancies. However, it remains unclear how NC interacts with STAT3, ERK or hedgehog signaling and deactivate it, although we demonstrate these multiple signaling pathways are affected by NC. It is unknown whether NC is a direct transcriptional suppressor of STAT3, ERK, SHH or Gli. In addition, the dose of NC used in *in vitro* study is as high as 50 μM. The concentration of NC certainly should be much lower if we try to demonstrate its druggability in pharmaceutical research. These intriguing questions must be addressed in future studies before NC can be further developed as a multi-target drug for cancer therapy.
